# Gene Expression Changes of Humans with Primary Mitral Regurgitation and Reduced Left Ventricular Ejection Fraction

**DOI:** 10.3390/ijms22073454

**Published:** 2021-03-26

**Authors:** Feng-Chun Tsai, Yu-Lin Chen, Kun-Chi Yen, Cheng-Hsun Chiu, Jui-Hsuan Chen, Yung-Hsin Yeh, Pei-Chien Tsai

**Affiliations:** 1 Division of Cardiac Surgery, Chang Gung Memorial Hospital, Taoyuan City 333, Taiwan; lutony@cgmh.org.tw; 2 School of Medicine, Chang Gung University, Taoyuan City 33302, Taiwan; dcsam999@gmail.com (K.-C.Y.); yys0tw@yahoo.ca (Y.-H.Y.); 3 Department of Biomedical Sciences, Chang Gung University, Taoyuan City 33302, Taiwan; cenachen46@gmail.com (Y.-L.C.); qswe81008@gmail.com (J.-H.C.); 4 Cardiovascular Department, Chang Gung Memorial Hospital, Taoyuan City 333, Taiwan; 5 Division of Pediatric Infectious Diseases, Department of Pediatrics, Chang Gung Memorial Hospital, Taoyuan City 333, Taiwan; chchiu@adm.cgmh.org.tw; 6 Molecular Infectious Disease Research Center, Chang Gung Memorial Hospital, Taoyuan City 333, Taiwan; 7 Graduate Institute of Biomedical Sciences, Chang Gung University, Taoyuan City 33302, Taiwan; 8 Healthy Aging Research Center, Chang Gung University, Taoyuan City 33302, Taiwan

**Keywords:** mitral regurgitation, cardiac fibrosis, reduced ejection fraction, transcriptome-wide association analysis

## Abstract

Patients with primary mitral regurgitation (MR) may remain asymptomatic for many years. For unknown reasons, some shift from a compensated to a decompensated state and progress to fatal heart failure. To elucidate the genetic determinants of this process, we recruited 28 patients who underwent mitral valve surgery and stratified them into control, compensated MR, and decompensated MR groups. Tissue biopsies were obtained from the patients’ left ventricular (LV) lateral wall for a transcriptome-wide profiling of 64,769 probes to identify differentially expressed genes (DEGs). Using cutoff values at the 1% FDR significance level and sex- and age-adjusted regression models, we identified 12 significant DEGs (*CTGF*, *MAP1B*, *SERPINE1*, *MYH9*, *MICAL2*, *MYO1D*, *CRY1*, *AQP7P3*, *HTRA1*, *PRSS23*, *IGFBP2*, and *FN1*). The most significant gene was *CTGF* (adjusted R^2^ = 0.74, *p* = 1.80 × 10^−8^). We found that the majority of genes expressed in the more advanced decompensated MR group were pro-fibrotic genes associated with cardiac fibrosis. In particular, six pro-fibrotic genes (*CTGF*, *SERPINE1*, *MYH9*, *HTRA1*, *PRSS23*, and *FN1*) were overexpressed and enriched in pathways involved in ECM (extracellular matrix) protein remodeling. Therapeutic interventions that antagonize these six genes may slow the progression toward decompensated MR.

## 1. Introduction

Primary mitral regurgitation (MR) is an abnormality of the mitral valve, most commonly due to myxomatous degeneration of the valve leaflets [1]. This prevents the valve from closing completely, resulting in retrograde blood flow from the left ventricle into the left atrium. During the compensatory phase of the disease, the LV dilates to maintain normal wall stress and pressure [1]. As the disease progresses, the ventricle exhibits more spherical shape, further increasing the LV end-diastolic pressure. At the same time, the contractile state of the myocardium decreases under the combined effect of myofiber reduction and interstitial fibrosis. The decompensated phase of the disease occurs when irreversible LV dysfunction develops with symptoms of heart failure [1].

Little is known about the molecular mechanisms that cause irreversible LV dysfunction in decompensated MR. Only recently, gene expression has been compared between individuals with compensated and decompensated MR using a panel of 109 candidate genes selected from animal and human studies [2]. The transition to decompensated MR is characterized by increased expression of *NPPA*, *NPPB*, inflammatory, ECM regulatory, and apoptotic genes, as well as aberrant expression of calcium regulatory and mitochondrial function genes [2].

The aim of this study was to perform whole transcriptome profiling of patients with compensated and decompensated MR. Subsequently, differentially expressed genes (DEGs) were analyzed by Gene Ontology (GO) and Kyoto Encyclopedia of Genes and Genomes (KEGG) analysis to identify pathological pathways in patients with compensated and decompensated MR.

## 2. Results

### 2.1. Study Participants

The baseline characteristics of the 28 patients are shown in Table 1. Patients were divided into three groups: control, compensated MR group, and decompensated MR group. In the analysis of LV dilatation, the control and CMR groups were combined and analyzed because there were significant differences between the control and CMR groups in LV end-diastolic diameter (LVEDD) and LV end-systolic diameter (LVESD), which were larger in the CMR group than in the control group (*p* = 5.86 × 10^−4^ and *p* = 0.049, respectively), but there were no significant differences in shortening fraction (SF) or ejection fraction (EF) between the two groups. The DMR group was not included in the analysis of LV dilatation because of the significantly lower EF in the DMR group. In contrast, SF and EF were significantly lower in the DMR group than in the control and CMR groups (*p* = 3.30 × 10^−4^ and 2.49 × 10^−5^, respectively), whereas LVEDD and LVESD were not significantly different from the other two groups (except for LVESD in DMR versus CMR, *p* = 0.024). In the analysis of LV dysfunction due to reduced EF, the DMR, CMR, and control groups were combined.

### 2.2. Identification of DEGs Associated with LV Dilatation and LV Dysfunction

**Figure 1:**
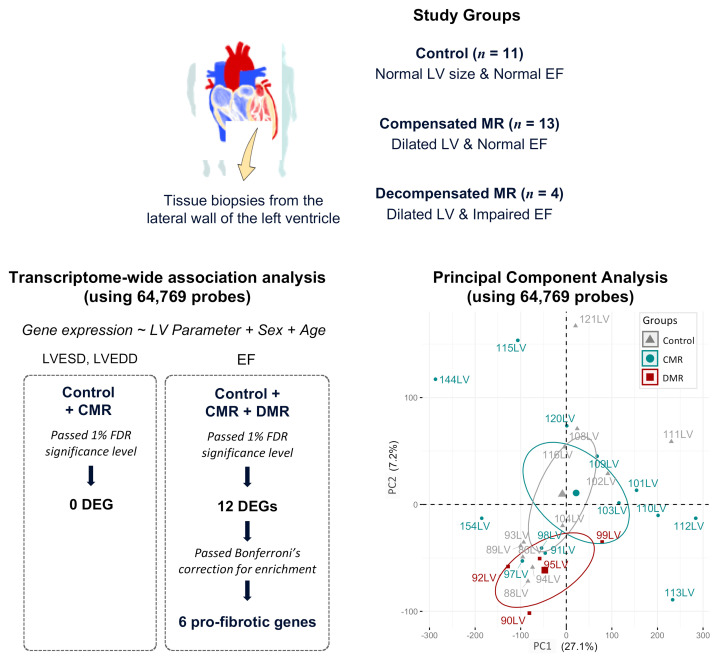
Summary of workflow and main findings.

A transcriptome-wide association analysis was performed on 64,769 probes covering 25,100 annotated human genes, adjusted for sex and age as covariates (Figure 1). The first and second principal components (PCs) jointly explained 34.3% of the total variance. Compensated MR and controls were similar to each other on PC1 and PC2, while decompensated MR was separate from compensated MR and controls on PC2.

**Figure 2:**
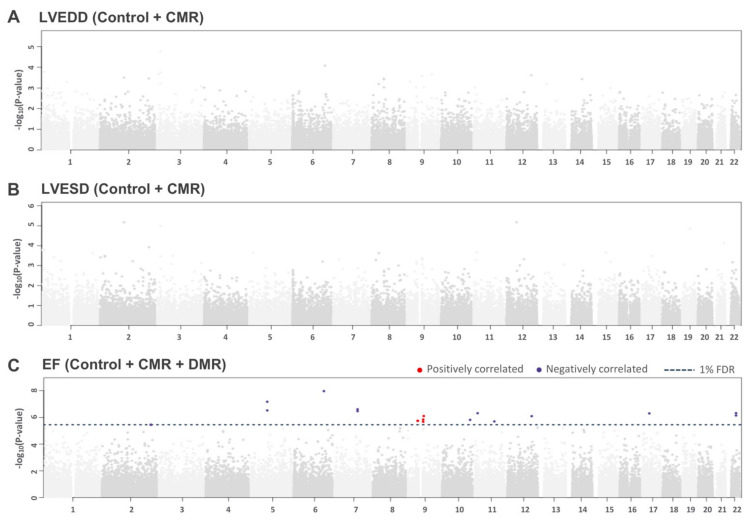
Transcriptome-wide association analysis. (**A**) LVEDD, (**B**) LVESD, (**C**) Ejection fraction. Each data point on the Manhattan plot corresponds to a TWAS result of −log_10_ of the *p*-value. The blue dashed line corresponds to the transcriptome-wide significance threshold for 1% FDR, and for ejection fraction, the significance threshold is at *p* = 3.56 × 10^−6^. Positive correlation probes are in red and negative correlation probes are in blue.

When using the LVEDD and LVESD parameters to compare gene expression changes in patients with normal and dilated LV, we did not find DEGs that passed the 1% FDR significance level (Figure 2A,B). However, after combining the three groups, 19 probes passed the 1% FDR significance level and were associated with reduced EF (Figure 2C). Four probes were positively correlated (i.e., positive β coefficients) and 15 probes were negatively correlated. Of these probes, 12 significant DEGs were located in the coding region of the transcribed genes, one of which was positively correlated with EF and the other 11 were negatively correlated with EF.

Table 2 shows the the top suggestive DEGs associated with increased LVEDD and LVESD including probe set IDs, chromosomes, start and end base pairs (GRCh37/hg19), symbols of transcribed genes and their descriptions, followed by the 12 significant DEGs associated with reduced EF.

**Figure 3:**
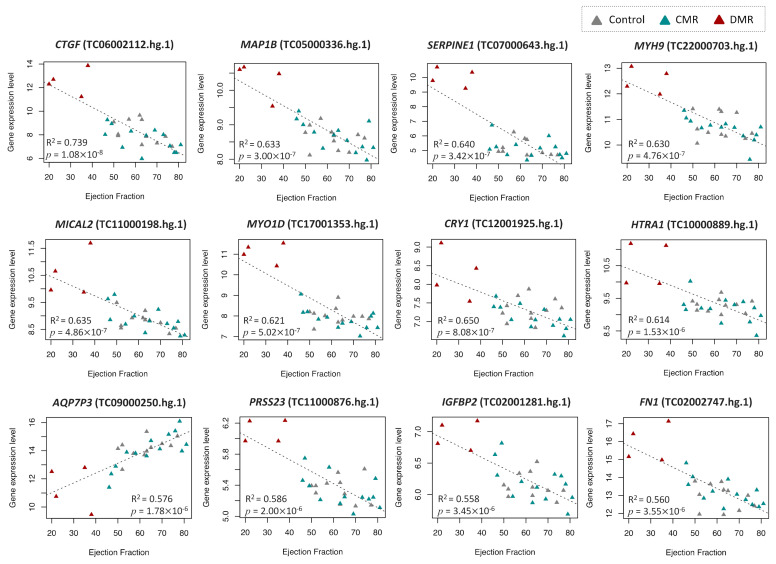
Correlation of gene expression levels with reduced EF in each of the 12 significant DEGs.

Figure 3 shows the direction of the β coefficient correlation values for each of the 12 significant DEGs. The expression levels of all genes increased with reduced EF, and only the expression of *AQP7P3* decreased with reduced EF.

### 2.3. Functional and Pathway Enrichment Analysis

**Figure 4:**
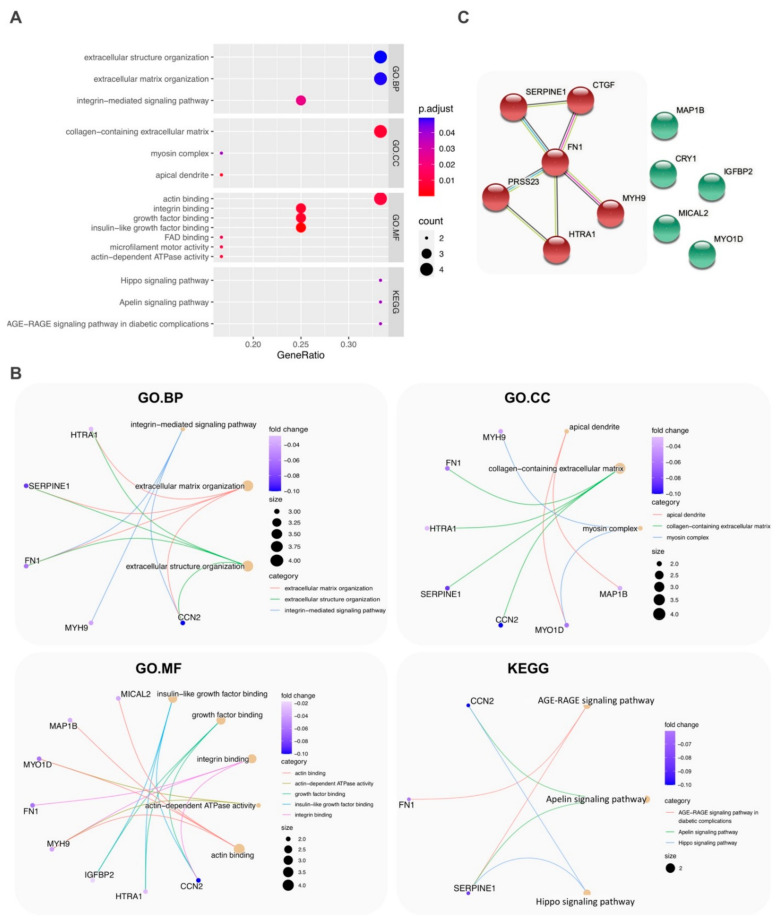
Enriched GO terms and KEGG pathways for the 12 significant DEGs. (**A**) GO terms and pathways that passed Bonferroni correction *p* < 0.05 and *q* < 0.05. (**B**) Relationships between mRNA and GO terms in BP (biological process), CC (cellular component), MF (molecular function), and KEGG pathways; fold change are β coefficient correlation values (**C**) STRING network showing the highest interaction enrichment between *CTGF*, *SERPINE1*, *FN1*, *PRSS23*, *HTRA1*, and *MYH9* genes (PPI enrichment *p* value 1.8 × 10^−4^).

Enrichment of GO and KEGG pathways was performed for the 12 significant DEGs. Figure 4A shows that for the GO.BP, the relevant terms were extracellular structure organization (GO:0043062), extracellular matrix organization (GO:0030198), and integrin-mediated signaling pathway (GO:0007229). For the GO.CC, they were collagen-containing extracellular matrix (GO:0062023). For the GO.MF, they were actin binding (GO:0003779), integrin binding (GO:0008305), growth factor binding (GO:0019838), and insulin-like growth factor binding (GO:0031994). For the KEGG pathway, they were the hippo signaling pathway (hsa04390), apelin signaling pathways (hsa04371), and AGE-RAGE signaling pathway in diabetic complications (hsa04933).

Figure 4B is a conceptual network diagram of genes showing the relationship between mRNA and enrichment pathways. Examples of the most frequently co-occurring genes lying in the same pathways were *CTGF*/*SERPINE1* and *MHY9*/*MYO1D*. Figure 4C shows the STRING network using 11 genes (*AQP7P3* was omitted as it was not searchable in the STRING database). The network revealed that the highest interaction enrichment was between six genes.

## 3. Discussion

Our transcriptome-wide profiling revealed that the majority of genes expressed during the transition to decompensated MR are pro-fibrotic genes. Cardiac fibrosis in the context of valvular disease refers to the proliferation of interstitial fibrosis involving the deposition of collagen-rich ECM in the interstitial space between cells in response to abnormal pressure loads [3]. Short-term interstitial fibrosis can be adaptive, but sustained activation of fibrotic pathways causes excessive accumulation of ECM and disruption of tissue function. The compensated MR group showed only suggestive changes in gene expression compared to the control group. Regarding the top genes associated with LV dilatation, we observed decreased expression of *FHL2*, whose overexpression increases ECM production and interstitial fibrosis [4], and increased expression of *NPPB*, which has been shown to be a cardiomyocyte-derived antifibrotic factor in ventricular remodeling [5]. Increased expression of *LINC01276*, a non-coding RNA gene, which has not been previously reported with increased LV dilatation, was also observed.

For LV dysfunction-related decompensated MR, 12 significant DEGs (*CTGF*, *MAP1B*, *SERPINE1*, *MYH9*, *MICAL2*, *MYO1D*, *CRY1*, *AQP7P3*, *HTRA1*, *PRSS23*, *IGFBP2* and *FN1*) passed the 1% FDR significance threshold. *CTGF* (encoding the CCN2 protein) was the most significantly transcribed gene in this study (*p* = 1.80 × 10^−8^). It connects with other genes involved in extracellular structural organization, ECM organization, integrin-mediated pathway, collagen-containing extracellular matrix, integrin binding, growth factor binding, insulin-like growth factor binding, Apelin signaling pathway and Hippo signaling pathway. *CTGF* is a well-known central mediator of fibrosis that activates myofibroblast and ECM protein remodeling. It is also significantly expressed in human myxomatous mitral valves compared to non-myxomatous mitral valves [6].

Five other pro-fibrotic genes in the STRING network are associated with *CTGF*. They are *SERPINE1* that encodes PAI (plasminogen activator inhibitor)-1, whose loss-of-function mutations are associated with human cardiac fibrosis [7]. *FN1* participates in ECM remodeling and its inhibition attenuates fibrosis and improves heart failure [8]. *HTRA1* reduces cellular secretion of mature TGF-β1, and CTGF synergizes with TGF-β to induce ECM deposition [9]. *PRSS23* is an activator of endothelial-to-mesenchymal transition and contributes to increased ECM protein production by cardiac fibroblasts, so exacerbating cardiac fibrosis [10]. *MYH9* inhibition suppresses TGF-β1-induced differentiation of lung fibroblast to myofibroblast [11].

TGF-β also plays a crucial role in fibrosis and is released in the pressure-overloaded heart in addition to neurohumoral mediators such as angiotensin, aldosterone, and norepinephrine [12]. The TGF-β pathway is controlled by ubiquitin-modifying enzymes and dysregulated ubiquitination is implicated in the deterioration of LV function [13]. So far, antagonizing TGF-β in heart failure has been toxic to humans [3]. However, treatment of mice with monoclonal antibodies to CTGF revealed encouraging results with reduced LV mass, cardiomyocyte hypertrophy, and myocardial fibrosis [14]. We suggest that the pro-fibrotic genes identified in this study have the potential to be therapeutic targets.

The remaining genes in the STRING network are associated with decompensated MR, but may not be directly related to fibrosis. They include *MAP1B*, which regulates the organization of the cytoskeleton [15]. *MICAL2* affects the regeneration of myofilaments and muscle tissue [16]. *MYO1D* is expressed in podocytes [17]. *CRY1* is a human circadian clock gene; and acute myocardial infarction and arrythmias are regulated by circadian clock genes [18]. *AQP7P3* is the main aquaglyceroporin in the heart situated in between the capillary lumen and interstitial space that acts as a glycerol facilitator, and its dysfunction can lead to cardiac hypertrophy and death [19]. Finally, elevated *IGFBP2* inhibits IGF-1, which regulates LV dysfunction and is a biomarker for predicting heart failure [20].

The present study has several limitations. First, it is a cross-sectional study that precludes a causal relationship. Second, MR is a progressive disease, and LV remodeling becomes advanced when the duration of MR is prolonged. We did not provide detailed information on disease duration and severity, because our review of patient records revealed that most patients had undergone mitral valve surgery for less than six months upon the diagnosis of severe MR. Some of the patients were referred from other hospitals, which resulted in a missing or slightly unreliable assessment of the duration of severe MR in the referred patients. In addition, the time point between moderate and severe MR may be defined rather arbitrarily as MR progresses over time. We think that for some patients, LV remodeling may be already underway when cardiac echography shows that the LV is already dilated, even if MR is still in the moderate phase. Considering the heterogeneous course of progression of MR-induced LV remodeling and the missing or unreliable documentation of the diagnosis of severe MR, we think that the duration of MR does not affect the results of this study.

## 4. Materials and Methods

### 4.1. Data Collection

We recruited 28 unrelated patients; 11 of whom had normal LV diameter and preserved ejection fraction, most of whom had rheumatic disease with mitral stenosis; 13 subjects had primary MR with dilated LV and preserved ejection fraction; and 4 subjects had dilated LV with reduced ejection fraction. The study subjects were recruited between December 2013 and December 2018 at Chang Gung Memorial Hospital in Linkou, Taiwan. The study protocol complied with the ethical guidelines of the 1975 Declaration of Helsinki and was approved by the Chang-Gung Medical Foundation Institutional Review Board (IRB No: 20170824B0, approved on 14 June 2017; 100-4076A3, approved on 31 January 2012; 103-7141C, approved on 13 January 2015). Written informed consent was obtained from all participants. All patients underwent mitral valve correction surgery during which LV tissue was collected from the lateral endocardial wall. We excluded from this study patients with recent myocardial infarction, stroke, acute heart failure, and major valvular heart disease. Detailed baseline information, such as age, gender, disease history, previous Maze procedure history, rheumatic status and clinical measurements (LVEDD, LVESD, SF, EF) were collected from their electronic hospital records.

### 4.2. RNA Isolation and Microarrays

RNA samples were extracted from fresh LV tissue after cardiac surgery. Total RNA was extracted using Trizol reagent (Invitrogen, Carlsbad, CA, USA), purified and concentrated with WelPrep tissue RNA kit (Welgene, Taipei, Taiwan). RNA quality was assessed using an Agilent Bioanalyzer 2100 (Agilent Technologies, Santa Clara, CA, USA). Gene expression profiles in tissue RNA were analyzed using the human U133A (Clariom D) GeneChip (Affymetrix, Santa Clara, CA, USA) according to the manufacturer’s protocol. Expression data were evaluated by Gene Level-SST RMA of Applied Biosystems Transcriptome Analysis Console (TAC).

### 4.3. Statistical Analysis

Clinical characteristics between two independent groups were compared using Student’s *t*-test. Sex ratios were calculated using Fisher’s exact test. Principal component analysis was used to identify potential covariates to be included in statistical models for analysis and to demonstrate clustering of patients. Transcriptome-wide association analysis was performed for each probe using a linear regression model, adjusting for age and sex. A false discovery rate (FDR) of 1% was set as the significance threshold using the Benjamini-Hochberg method in R package qvalue [21]. GO and KEGG enrichment of DEGs was performed using hypergeometric analysis and Bonferroni correction with *p* < 0.05 set as the significance level in R package clusterProfiler [22]. STRING v11.0 http://string-db.org (accessed in 7 March 2021) [23].

## 5. Conclusions

We identified the molecular components in decompensated MR as being due to the overexpression of pro-fibrotic genes. Further animal models and human cardiomyocyte cultures are needed to validate their effects on cardiac fibrosis.

## Figures and Tables

**Table 1:** Clinical characteristics of 28 patients referred for mitral valve correction surgery.

Clinical Variable	Control *n* = 11	CMR *n* = 13	DMR *n* = 4	*p* Value (CMR vs. Control)	*p* Value (DMR vs. Control)	*p* Value (DMR vs. CMR)
Age, years	62.45 ± 11.34	59.46 ± 12.87	59.75 ± 13.96	0.551	0.555	0.970
Sex (*n*, %)						
Female	7 (64)	3 (23)	2 (50)	0.0953	0.999	0.5378
Male	4 (36)	10 (77)	2 (50)			
Parameters for LV diameter						
LVEDD, mm	47.27 ± 4.03	57.85 ± 8.07	61.25 ± 14.93	5.86 × 10^−4^	0.157	0.554
LVESD, mm	31.18 ± 4.94	37.69 ± 9.27	54.00 ± 17.38	0.049	0.077	0.024
Parameters for LV function						
SF, %	0.34 ± 0.07	0.35 ± 0.10	0.13 ± 0.10	0.767	3.30 × 10^−4^	1.20 × 10^−3^
EF, %	62.27 ± 9.01	64.46 ± 12.69	28.75 ± 9.07	0.637	2.49 × 10^−5^	1.12 × 10^−4^

Values are mean ± SD in all variables except for gender; *n*, individuals; CMR, compensated MR; DMR, decompensated MR; LVEDD, left ventricular end diastolic diameter; LVESD, left ventricular end systolic diameter; SF, shortening fraction; EF, ejection fraction. *p*-Values for continuous variables were calculated by Student’s *t*-test followed F test of equality of variances; *p*-Values for gender differences were calculated by Fisher’s exact test.

**Table 2:** Top DEGs associated with LVEDD, LVESD, and EF parameters.

Probe Set ID	CHR	Start	End	β Coef.	S.E.	*p* Value	Gene Symbol	Description
**LVEDD**								
TC03001196.hg.1	3	14389951	14394068	0.025	0.004	1.70 × 10^−5^	*LINC01267*	long intergenic non-protein coding RNA 1267
TC01002205.hg.1	1	11917521	11918992	0.298	0.065	1.68 × 10^−4^	*NPPB*	natriuretic peptide precursor B
**LVESD**								
TC02002167.hg.1	2	105974169	106055230	−0.037	0.006	6.67 × 10^−6^	*FHL2*	four and a half LIM domains 2
TC03001196.hg.1	3	14389951	14394068	0.025	0.004	1.01 × 10^−5^	*LINC01267*	long intergenic non-protein coding RNA 1267
**EF**								
TC06002112.hg.1	6	132269316	132272518	−0.100	0.012	1.80 × 10^−8^	*CTGF*	connective tissue growth factor
TC05000336.hg.1	5	71403061	71505397	−0.036	0.005	3.00 × 10^−7^	*MAP1B*	microtubule associated protein 1B
TC07000643.hg.1	7	100770370	100782547	−0.092	0.013	3.43 × 10^−7^	*SERPINE1*	serpin peptidase inhibitor, clade E (nexin, plasminogen activator inhibitor type 1), member 1
TC22000703.hg.1	22	36677323	36784063	−0.040	0.006	4.76 × 10^−7^	*MYH9*	myosin, heavy chain 9, non-muscle
TC11000198.hg.1	11	12115543	12285332	−0.038	0.006	4.86 × 10^−7^	*MICAL2*	microtubule associated monooxygenase, calponin and LIM domain containing 2
TC17001353.hg.1	17	30819627	31203902	−0.061	0.009	5.20 × 10^−7^	*MYO1D*	myosin ID
TC12001925.hg.1	12	107385142	107487607	−0.025	0.004	8.08 × 10^−7^	*CRY1*	cryptochrome circadian clock 1
TC10000889.hg.1	10	124221041	124274424	−0.029	0.005	1.53 × 10^−6^	*HTRA1*	HtrA serine peptidase 1
TC09000250.hg.1	9	42858152	42893137	0.072	0.011	1.78 × 10^−6^	*AQP7P3*	aquaporin 7 pseudogene 3
TC11000876.hg.1	11	86511282	86663886	−0.016	0.003	2.00 × 10^−6^	*PRSS23*	protease, serine, 23
TC02001281.hg.1	2	217497551	217529159	−0.018	0.003	3.54 × 10^−6^	*IGFBP2*	insulin like growth factor binding protein 2
TC02002747.hg.1	2	216225163	216300895	−0.060	0.010	3.55 × 10^−6^	*FN1*	fibronectin 1

CHR, chromosome; β coef., β coefficients; S.E., standard error. P-value were evaluated by linear regression model adjusted for age and gender.

## Data Availability

The data presented in this study are available from the corresponding authors upon request.

## Abbreviations

FDR	False discovery rate
LV	Left ventricle
MR	Mitral regurgitation
ECM	Extracellular matrix
LVEDD	Left ventricular end-diastolic diameter
LVESD	Left ventricular end-systolic diameter
SF	Shortening fraction
EF	Ejection fraction

## References

[1] El Sabbagh A., Reddy Y.N.V., Nishimura R.A. (2018). Mitral Valve Regurgitation in the Contemporary Era: Insights Into Diagnosis, Management, and Future Directions. JACC Cardiovasc. Imaging.

[2] McCutcheon K., Dickens C., van Pelt J., Dix-Peek T., Grinter S., McCutcheon L., Patel A., Hale M., Tsabedze N., Vachiat A. (2019). Dynamic Changes in the Molecular Signature of Adverse Left Ventricular Remodeling in Patients With Compensated and Decompensated Chronic Primary Mitral Regurgitation. Circ. Heart Fail..

[3] Sweeney M., Corden B., Cook S.A. (2020). Targeting cardiac fibrosis in heart failure with preserved ejection fraction: Mirage or miracle?. EMBO Mol. Med..

[4] Zhou S.G., Ma H.J., Guo Z.Y., Zhang W., Yang X. (2018). FHL2 participates in renal interstitial fibrosis by altering the phenotype of renal tubular epithelial cells via regulating the beta-catenin pathway. Eur. Rev. Med. Pharmacol. Sci..

[5] Tamura N., Ogawa Y., Chusho H., Nakamura K., Nakao K., Suda M., Kasahara M., Hashimoto R., Katsuura G., Mukoyama M. (2000). Cardiac fibrosis in mice lacking brain natriuretic peptide. Proc. Natl. Acad. Sci. USA.

[6] Hagler M.A., Hadley T.M., Zhang H., Mehra K., Roos C.M., Schaff H.V., Suri R.M., Miller J.D. (2013). TGF-beta signalling and reactive oxygen species drive fibrosis and matrix remodelling in myxomatous mitral valves. Cardiovasc. Res..

[7] Iwaki T., Urano T., Umemura K. (2012). PAI-1, progress in understanding the clinical problem and its aetiology. Br. J. Haematol..

[8] Valiente-Alandi I., Potter S.J., Salvador A.M., Schafer A.E., Schips T., Carrillo-Salinas F., Gibson A.M., Nieman M.L., Perkins C., Sargent M.A. (2018). Inhibiting Fibronectin Attenuates Fibrosis and Improves Cardiac Function in a Model of Heart Failure. Circulation.

[9] Muratoglu S.C., Belgrave S., Hampton B., Migliorini M., Coksaygan T., Chen L., Mikhailenko I., Strickland D.K. (2013). LRP1 protects the vasculature by regulating levels of connective tissue growth factor and HtrA1. Arterioscler. Thromb. Vasc. Biol..

[10] Bayoumi A.S., Teoh J.P., Aonuma T., Yuan Z., Ruan X., Tang Y., Su H., Weintraub N.L., Kim I.M. (2017). MicroRNA-532 protects the heart in acute myocardial infarction, and represses prss23, a positive regulator of endothelial-to-mesenchymal transition. Cardiovasc. Res..

[11] Sun X., Zhu M., Chen X., Jiang X. (2020). MYH9 Inhibition Suppresses TGF-beta1-Stimulated Lung Fibroblast-to-Myofibroblast Differentiation. Front. Pharmacol..

[12] Frangogiannis N.G. (2019). The Extracellular Matrix in Ischemic and Nonischemic Heart Failure. Circ. Res..

[13] Tsai F.C., Chang G.J., Lai Y.J., Chang S.H., Chen W.J., Yeh Y.H. (2020). Ubiquitin Pathway Is Associated with Worsening Left Ventricle Function after Mitral Valve Repair: A Global Gene Expression Study. Int. J. Mol. Sci..

[14] Vainio L.E., Szabo Z., Lin R., Ulvila J., Yrjola R., Alakoski T., Piuhola J., Koch W.J., Ruskoaho H., Fouse S.D. (2019). Connective Tissue Growth Factor Inhibition Enhances Cardiac Repair and Limits Fibrosis After Myocardial Infarction. JACC Basic Transl. Sci..

[15] Montenegro-Venegas C., Tortosa E., Rosso S., Peretti D., Bollati F., Bisbal M., Jausoro I., Avila J., Caceres A., Gonzalez-Billault C. (2010). MAP1B regulates axonal development by modulating Rho-GTPase Rac1 activity. Mol. Biol. Cell.

[16] Giarratana N., Conti F., La Rovere R., Gijsbers R., Carai P., Duelen R., Vervliet T., Bultynck G., Ronzoni F., Piciotti R. (2020). MICAL2 is essential for myogenic lineage commitment. Cell Death Dis..

[17] Brunskill E.W., Georgas K., Rumballe B., Little M.H., Potter S.S. (2011). Defining the molecular character of the developing and adult kidney podocyte. PLoS ONE.

[18] Rabinovich-Nikitin I., Lieberman B., Martino T.A., Kirshenbaum L.A. (2019). Circadian-Regulated Cell Death in Cardiovascular Diseases. Circulation.

[19] Verkerk A.O., Lodder E.M., Wilders R. (2019). Aquaporin Channels in the Heart-Physiology and Pathophysiology. Int. J. Mol. Sci..

[20] Barutaut M., Fournier P., Peacock W.F., Evaristi M.F., Caubere C., Turkieh A., Desmoulin F., Eurlings L.W.M., van Wijk S., Rocca H.B. (2020). Insulin-like Growth Factor Binding Protein 2 predicts mortality risk in heart failure. Int. J. Cardiol..

[21] Storey J.D., Taylor J.E., Siegmund D. (2004). Strong control, conservative point estimation and simultaneous conservative consistency of false discovery rates: A unified approach. J. R. Stat. Soc. Ser. B Stat. Methodol..

[22] Yu G., Wang L.G., Han Y., He Q.Y. (2012). clusterProfiler: An R package for comparing biological themes among gene clusters. OMICS J. Integr. Biol..

[23] Szklarczyk D., Franceschini A., Wyder S., Forslund K., Heller D., Huerta-Cepas J., Simonovic M., Roth A., Santos A., Tsafou K.P. (2015). STRING v10: Protein-protein interaction networks, integrated over the tree of life. Nucleic Acids Res..

